# A thermodynamic model showing that information recording can drive active ion transport

**DOI:** 10.1038/s41392-023-01361-3

**Published:** 2023-04-14

**Authors:** Xiang Zou, Kun Song, Minbiao Ji, Lingzhao Min, Liangfu Zhou, Ying Mao, Liang Chen

**Affiliations:** 1 Department of Neurosurgery, Neurosurgical Institute of Fudan University, National Center for Neurological Disorders, Huashan Hospital, Fudan University, Shanghai, 200040 China; 2 Department of Physics, Fudan University, Shanghai, 200433 China; 3 State Key Laboratory of Medical Neurobiology and MOE Frontiers Center for Brain Science, School of Basic Medical Sciences and Institutes of Brain Science, Fudan University, Shanghai, 200040 China

**Keywords:** Biophysics, Structural biology

**Dear Editor**,

Ion gradients are characteristic features of a living cell that do not represent thermodynamic equilibrium. These unequilibrium status are maintained by various ion pumps such as Na-K-ATPase, which are known as the P-type ATPases. Working mechanism of Na-K-ATPase is described as Post–Albers cycle^1^ (Fig. 1a). However, the reaction scheme is non-Michaelis–Menten kinetics assembled from transient-state results, which can hardly be used to rebuild the real reaction cycle. Here we introduce a thermodynamic model for active ion transport that describes the pump acting as a ‘Maxwell’s demon’,^2^ which has come to refer to any situation in which a rectification of microscopic fluctuations produces a decrease in thermodynamic entropy.^3^ Recent progress in physics has already proven the possibility of a Maxwell’s demon that does not use its own intelligence but rather streams of information, which can be regarded as ATP to ADP flip and generalized to build up kinetic equation for such P-type ATPases.

**Fig. 1:**
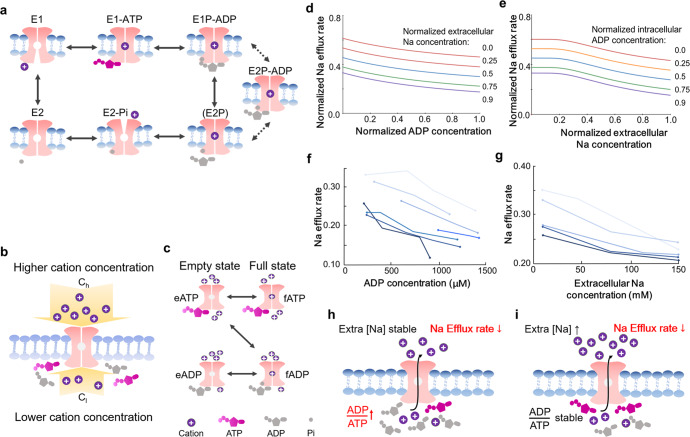
Working mechanism of an active ion transport pump and Na-K exchange rate affected by the intracellular ADP or extracellular Na concentration. **a** Catalytic cycle of P-type ATPases exemplified by a cation transport.^5^ E1: high-affinity state for cation, E2: low-affinity state for cation, P: phosphorylated state. **b** Pump framework that can interact with nucleotides and exchange ions with either the lower- or higher-concentration side. **c** The pump performs intrinsic transitions mediated by the higher-concentration side (horizontal arrows). The pump and a nucleotide perform cooperative transitions eATP→fADP mediated by the lower-concentration side (diagonal arrow). **d** Na efflux rate controlled by increasing ADP concentration at various normalized preset intracellular Na concentrations. **e** Decreased Na efflux rate after the extracellular Na increasing at various normalized preset intracellular ADP concentrations. **f** The effect of various intracellular ADP levels on ouabain-sensitive Na-K exchange in resealed ghosts. **g** The stimulation percentage of ouabain-sensitive Na efflux after external Na increasing. The phosphoarginine regenerating system was used to set and maintain ADP and ATP concentrations. See supplementary methods. **h**, **i** Illustrations of these two effects

Here we introduce an ion pump with a duplex channel controlled by a certain mechanism and an ATP or ADP binding site located in the inner matrix. The outer and inner matrix ion concentrations are defined as *C*_*h*_ and *C*_*l*_, with *C*_*h*_ > *C*_*l*_ (Fig. 1b). The pump will play the role of Maxwell’s demon as described below.

The pump is a two-state system, with states ‘f’ and ‘e’ characterized by the presence and absence of ions. The pump makes random transitions between these two states by exchanging ions with the outer matrix (higher ion concentration), as illustrated by the horizontal arrows in Fig. 1c. We will refer to these as intrinsic transitions that happen only rely on the parameters of outer matrix, including the ion concentration and binding ability. The corresponding transition rates R meet the requirements of the balance below:

1 $$\frac{{R_{e \to f}}}{{R_{f \to e}}} = e^{ - \frac{1}{{k_hC_h}}}$$

where *k*_*h*_ is a constant vary among pump types (mol^−1^). Transition rates R_e→f_ means the pump is occupied by the ion from outer matrix, while R_f→e_ means the ion is released from the pump to outer matrix. On the other hand, the pump and one ATP or ADP can make cooperative transitions: if the ATP binds the pump in state ‘e’, then the pump can simultaneously flip to state f with ATP → ADP, and vice versa (Fig. 1c, diagonal arrows). We will use the notation eATP↔fADP to denote these transitions, which are accompanied by an exchange of ions with the inner matrix. The corresponding transition rates again satisfy the following balance:

2 $$\frac{{R_{{{{\mathrm{eATP}}}} \to {{{\mathrm{fADP}}}}}}}{{R_{{{{\mathrm{fADP}}}} \to {{{\mathrm{eATP}}}}}}} = e^{ - \frac{1}{{k_lC_l}}}$$

where *k*_*l*_ is also a constant vary among pump types (mol^−1^). For later convenience, we also define

3 $${\it{\epsilon }} = \tanh \frac{{(C_h - C_l)}}{{2k_hk_lC_hC_l}}$$

whose value, 0 < *ϵ* < 1, quantifies the difference in ion concentration between the outer and inner matrix.

Finally, we assume that the inner matrix contains a mixture of ATP and ADP with proportions *p*_*t*_ and *p*_*d*_, respectively. Let

4 $$\begin{array}{*{20}{c}} {\delta \equiv p_t - p_d} \end{array}$$

denote the proportional excess of ATP.

We thus have the following dynamics. When ATP or ADP arrives at the pump, it interacts with binding site for a short interval then releases; the pump might make the diagonal transition shown in Fig. 1c and thus effect an ion exchange between the matrixes. The parameter ϵ defines the difference between intrinsic and cooperative transition rates of ion pump.

Assuming that each incoming nucleotide is an ATP. The pump and nucleotide then evolve together as shown in Fig. 1c. If the joint state at the end of the interaction interval is eATP or fATP, then it must be the case that every transition eATP → fADP was balanced by the opposite transition. If the final state is eADP or fADP, then we know that one ion was absorbed from the lower-concentration side, and a record of this process is imprinted in the nucleotide. Since the pump also exchanges ions with the higher-concentration side, we obtain a net ions efflux in the long run. More generally, if the incoming nucleotides are a mixture of ATP and ADP, then an excess of ATP (*δ* > 0) induces a statistical bias that favors the ion efflux, while an excess of ADP (*δ* < 0) produces inflow.

As described above, the transport process in this model reduces the entropy without consuming energy. Once the pump has reached steady state, let $p_t^\prime$ and $p_d^\prime$ denote the fractions of ATP and ADP in the released nucleotides, and let $\delta ^\prime = p_t^\prime - p_d^\prime$ denote the excess of released ATP. Then,

5 $${{{\Phi}}} \equiv p_d^\prime - p_d = \frac{{\delta - \delta ^\prime }}{2}$$

represents the fraction of an ADP molecule produced on average per interaction interval in the released nucleotide stream relative to the incoming binding stream. Since each ATP → ADP transition is accompanied by the transfer of ions from the lower-concentration side (Fig. 1c), the average number of ions net efflux per interaction is given by

6 $$Q_{l \to h} = {{{\Phi}}}$$

A positive value of *Q*_*l→h*_ indicates that the pump transfers ions against the concentration gradient.

To quantify the information-processing capability of the pump, we calculate the Shannon entropy, let

7 $$S\left( \delta \right) = - p_t\log p_t - p_d\log p_d = - \frac{{1 - \delta }}{2}\log \frac{{1 - \delta }}{2} - \frac{{1 + \delta }}{2}\log \frac{{1 + \delta }}{2}$$

denote the information content per nucleotide of the incoming nucleotide stream, and define *S*(*δ*^′^) by the same equation for the outgoing bit (nucleotide) stream. Then,

8 $${\Delta}S \equiv S\left( {\delta ^\prime } \right) - S\left( \delta \right) = S\left( {\delta - 2{{{\Phi}}}} \right) - S\left( \delta \right)$$

provides a measure of the extent by which the pump increases the information content of the ATP/ADP pool. A positive value of ∆*S* indicates that the pump writes information to the ATP/ADP pool, while a negative value indicates erasure.

As shown above, ${{{\Phi}}}$ determines both *Q*_*l→h*_ and ∆*S*. As previously described,^4^ under the established dynamics, the pump reaches a periodic steady state determined by the model parameters Λ ≡ (*δ, k, C*_*h*_*, C*_*l*_) in which

9 $${{{\Phi}}}\left( {\Lambda} \right) = \frac{{\delta - {\it{\epsilon }}}}{2}\eta \left( {\Lambda} \right),\eta \,>\, 0$$

10 $$Q_{l \to h}\left( {\frac{{C_h - C_l}}{{k_hk_lC_hC_l}}} \right) + {\Delta}S \ge 0$$

The crucial point is that the sign of ${{{\Phi}}}$ is the same as that of *δ* − *ϵ*. We can consider two effective forces: the bias induced by the incoming nucleotide stream, which favors ${{{\Phi}}}$ > 0 when *δ* > *ϵ*, and the concentration gradient, quantified by *ϵ*, which favors ${{{\Phi}}}$ < 0 when *ϵ* > *δ*. The direction of ion flow is determined by the difference *δ* *−* *ϵ*.

According to our hypothesis above, the transport ability of pumps has little relationship with the energy of nucleotides but is related to the concentration differences. For example, in Na-K-ATPase, we calculate ${{{\varPhi}}}$ in detail as

11 $${{{\Phi}}} = \frac{{\delta - \tan\!{{{\mathrm{h}}}}\frac{{C_h - C_l}}{{2k_hk_lC_hC_l}}}}{2}\left( {1 - e^{ - \left( {1 - \tanh\frac{1}{{2k_hC_h}}\tanh\frac{1}{{2k_lC_l}}} \right)}} \right)$$

The Na transfer rate $({{{\Phi}}})$ by increasing the intracellular ATP or ADP concentration at various normalized intracellular Na concentrations from 0.0 to 0.9 (ratio of intra/extracellular Na concentration). We found that the efflux rate was decreased with increasing ADP concentration at any extracellular Na concentrations (Fig. 1d). The efflux rate also decreases at various intracellular ATP/ADP concentrations with increasing extracellular Na concentration (Fig. 1e). Then, we reviewed the effect of varying the concentration of intracellular ADP at constant ATP on ouabain-sensitive Na/K exchange. We obtained an obvious negative correlation between the ouabain-sensitive flux and increasing ADP concentration (Fig. 1f). The initial Na efflux rate varied from 0.23 to 0.33 per hour, and decreased from 0.12 to 0.24 per hour when intracellular ADP concentration increased to 1500 μM. Similarly, inhibition of the ouabain-sensitive Na efflux by increasing extracellular Na concentration was also reviewed at a controlled ATP/ADP ratio. We obtained a negative correlation between ouabain-sensitive flux and increasing extracellular Na levels (Fig. 1g). Fig. 1h and i illustrates these two effects. Other simulations are shown in supplementary Fig. 1.

According to our theory, active ion transport in our framework is caused by only random ion motion with information recording rather than any other forces, which is quite different from traditional theory. The role of ATP to ADP transformation can be regarded as a 0 to 1 switch that increases Shannon entropy. In this case, ATP and ADP are not energy sources but information carriers. Thus, the energy released from high-energy phosphate bond in ATP can mainly become heat (thermal motion) to facilitate reaction (possibility of information recording), as well as overcome transmembrane potential. This understanding of the active ion transport system provides new perspective to coordination of different ion pumps in living cells. See supplementary discussion.

## Supplementary information

Supplementary Materials

## Data Availability

All data are available from the corresponding author on reasonable request.
