# Supplementary material for: A thermodynamic model showing that information recording can drive active ion transport
Source: Signal Transduct Target Ther. 2023 Apr 14;8:154. doi: 10.1038/s41392-023-01361-3 (PMC10101940; doi:10.1038/s41392-023-01361-3)
Supplement: Supplementary file 1 — Supplementary Materials [file 41392_2023_1361_MOESM1_ESM.docx]

Supplementary Materials for

A Thermodynamic Model Showing That Information Recording Can Drive Active Ion Transport

Xiang Zou^1,†^; Kun Song^1,†^; Minbiao Ji^3,†^; Lingzhao Min^1,†^; Liangfu Zhou^1^; Ying Mao^1,2,^*; Liang Chen^1,^*

1 Department of Neurosurgery, Neurosurgical Institute of Fudan University, National Center for Neurological Disorders, Huashan Hospital, Fudan University, Shanghai 200040, China

2 State Key Laboratory of Medical Neurobiology and MOE Frontiers Center for Brain Science, School of Basic Medical Sciences and Institutes of Brain Science, Fudan University, Shanghai 200040, China

3 Department of Physics, Fudan University, Shanghai 200433, China

†These authors contributed equally to this work.

*Corresponding Author: Ying Mao: [hs_maoying@vip.126.com](mailto:hs_maoying@vip.126.com); Liang Chen:[chenlianghs@126.com](mailto:chenlianghs@126.com)

**This PDF file includes:**

Supplementary Methods

Supplementary Results

Supplementary Discussions

Supplementary Fig. 1

Supplementary References

Supplementary Methods

The first series experiments to reveal the delicate balance between Na-K pump activation and cell energy currency, were performed in the red cell ghost system. In general, resealed red cell ghosts were made by procedures involving double haemolysis. The first haemolysis was carried out to reduce the cellular concentration of endogenous metabolites and adenylate kinase. Red blood cells were washed three times at 4°C by suspension and centrifugation (1 min at 12,000 g) with 5μl of solution containing 150 mM NaCl and 10 mM HEPES (pH 6.8 at 20°C). The packed cells at the end of the third washing (1 min at 12,000 g) were cooled in an ice bath and then hemolyzed by squirting 50 ml of cells into 3,500 ml of vigorously stirred hemolyzing medium (3 mM MgC12, 0.1 mM EDTA, and 5 mM HEPES, pH 6.5 at 0.3 ± 0.2° C). After 5 min, equal volumes of 3 M NaCl and 3 M KCl were added to bring the concentration of Na plus K in the hemolysis mixture to 150 mM. The pH was adjusted to 6.8 and the ghosts were resealed by incubating the suspension for 30 min at 37 °C. The second haemolysis introduced an adenylate kinase inhibitor together with adenine nucleotides and a regenerating system to maintain stable internal ATP and ADP levels. Creatine kinase was incorporated at the second hemolysis. Creatine phosphate was added and the ATP concentration was varied by incorporating different amounts at reversal. In order to manipulate ATP in the presence of appreciable levels of ADP and also to vary ADP itself, we incorporated excess arginine and phosphoarginine so that their concentrations will not change appreciably over the course of an experiment. By varying the phosphoarginine/arginine ratio, a range of different ADP/ATP ratios can be set and maintained. ^24^Na was added in a resealing process, and other preset concentrations of extracellular ions were adjusted. As noted, ^24^Na was mixed into the ghosts at reversal. Measurements of ^24^Na efflux were begun by adding 200 μl of a 50% ghost suspension to 6 ml of medium follow by water bath at 37°C. Samples were centrifugated and assayed for radioactivity in a gamma counter at -5, 15, 25, and 35 min. Ouabain-sensitive Na efflux was measured in the presence and absence of external K. The K-free medium contained 150 mM NaCl and 10 mM HEPES, pH 7 .4 with 10^-4^ M ouabain. In general, a single ghost preparation was divided into four equal fractions before reversal, with each fraction resealed to contain a different nucleotide mixture. Effluxes for each nucleotide condition were performed four times. The rate constant for Na efflux was determined from a least-squares linear fit of the log transform of the radioactivity remaining in the ghosts. The Na efflux rate was calculated by the following equation: $\ln\left( 1-{R_{s}}/{R_{eq}} \right)={}_{Na}^{o}{k\cdot t}$, where ${R_{s}}/{R_{eq}}$ represents the fraction of ^24^Na released over time, and ${}_{Na}^{o}k$ is the outward rate constant (per hour). Only data obtained between 5 and 35 min, where the loss followed a single exponential, were used in the determination of the rate constant ^1,2^.

Supplementary Results

In the case of Na-K-ATPase, potassium and sodium are exchanged in a set stoichiometry. We calculated the Na transfer rate ($\Phi$) by increasing the intracellular ATP or ADP concentration at various normalized intracellular Na concentrations (ratio of intra/extracellular Na concentration). We found that the Na efflux rate was increased by increasing the ATP concentration at various intracellular Na concentrations (supplementary Fig. 1a). In addition, the Na transfer rate can be increased by the removal of extracellular Na at all ADP concentrations, and the relative ADP concentration increasing can improve the effect induced by extracellular Na removal (supplementary Fig. 1b). Then, we reviewed the effect of varying intracellular ATP on the extent of ouabain-sensitive Na/K exchange in red cell ghosts. We found that the Na efflux could be considered a function of the ATP concentration; least-squares fitting the Na efflux against the ATP concentration yielded a curve that is very similar to a curve that we predicted (supplementary Fig. 1c). Moreover, the percent stimulation of ouabain-sensitive Na efflux caused by the removal of external Na was calculated by dividing the rate difference by the rate in the presence of Na. As internal ADP was increased, the percent stimulation correspondingly increased as expected (supplementary Fig. 1d). Supplementary Fig. 1e and f illustrates these two effects.

Supplementary Discussion

**Possibility of Less Cost: Recent Insight into Gradient Generation**

Related to the ion pump mechanism, J. C. Maxwell proposed a gedanken experiment known as ‘Maxwell’s demon’ in 1871^3^. In this thought experiment highlighting the statistical nature of the second law of thermodynamics, Maxwell imagined a tiny creature acting as a gatekeeper between two chambers filled with gases at different temperatures. By preferentially allowing fast-moving molecules to pass from the cold to the hot chamber and slow ones to pass in the other direction, this creature achieves refrigeration without the expenditure of energy. The term ‘Maxwell’s demon’ has come to refer not only to the original setting described by Maxwell but also more generally to any situation in which a rectification of microscopic fluctuations produces a decrease in thermodynamic entropy. In addition, recent progress in physics has already proven the possibility of a Maxwell’s demon that does not use its own intelligence but rather streams of information, such as a memory register sequence^4,5^. By this mechanism, the demon can transform the thermodynamic fluctuation to refrigeration or mechanical work. Recently, study quantum Maxwell’s demon has already been introduced with the ability of generating work from thermal excitations of a quantum spin via measurement and feedback control^6^. Similarly, ion pumps also seem to decrease the thermodynamic entropy. It is worth noting that all those ion transport events happen in nanoscale, in which the thermodynamic fluctuation cannot be ignored. Although ATP can supply enough Gibbs free energy to drive this reaction, we still care about the theoretical lower bound of the energy consumption, in consideration of the ‘Ockham's Razor’. In the following context, we will introduce a balance equation based on ion concentration and ATP/ADP ratio, from the perspective of Maxwell’s demon.

**Interpretation**

ATP is the universal energy currency in eukaryotic cells. Most of the biological processes in cells rely on the energy released from ATP to ADP transformation. ATP is mainly synthesized from ADP in mitochondria and released through ADP and ATP carriers to maintain a higher ATP concentration in the cytoplasm ^7^. It is generally considered that the maintenance of life requires negative entropy^8^. In a smaller scale, living cells must maintain an internal environment distinct from that of the extracellular matrix or a dynamic equilibrium for substance exchange, and allow action potential^9^. From a thermodynamic perspective, this process resists movement to thermal equilibrium by using negative entropy intake^10^. In this hypothesis, our active ion transport model first reveals the relationship between ATP consumption and Shannon entropy increasing, which can fit the entropy decrease during ion transport. The steady-state change in thermodynamic entropy due to the flow of ions, together with the change in information entropy per interaction interval, in which the information entropy of a random ATP and ADP stream is explicitly assigned the same thermodynamic status as the physical entropy associated with the transfer of ions. Thus, our model provides support for Landauer’s principle, which states that a thermodynamic cost must be paid for the erasure of memory. As in the living cells, mitochondria can play such a role for memory recovery under normal physiologic condition. Besides, ATPase will also play such a role as the converse Post–Albers cycle in certain circumstances.

According to our theory, questions raised previously can be roughly answered. First is the reversible Post–Albers cycle, which coincides with our model exactly. Reaction schemes for enzymes with non-Michaelis-Menten kinetics are generally assembled from transient-state results because there is no validated procedure to do this from steady state determinations. Transient-state studies provide very valuable information but are more difficult to perform, and they evaluate only partial reactions, which might not be the whole reaction cycle. Although there is no such specific reference to talk about this issue, the limitation is the consensus in biochemistry field^11^. A recent study on the P-type Ca^2+^ pump revealed that its transport process involves reversible steps, such as cation or ATP binding. However, the step following the release of ADP and extracellular release of Ca^2+^ is irreversible^12^. As supported by the demon’s behaviour in our work, this step is just related to the moment of information recording, which is irreversible when the bit stream passes by. According to our model, the throughput of transport closely depends on such variables as ion concentration, ATP proportion and temperature. In normal liquid condition, the ion-pump collision is more than one hundred times per second. As a result, the general ion transport efficiency can also reach hundreds of times under a high ATP proportion. Moreover, the high selectivity is just the intrinsic property of Maxwell’s demon, who has already been physically proven to act as an observer, without energy consumption. Therefore, the selectivity for ion pump is reasonable. In addition, the scheme in this study was designed only under a general circumstance, which could illustrate this model more clearly. Recently, Jiang et al. introduced the scheme of quantized chemical reaction during ATP-driven DNA replication^13,14^. They found multiple mid-infrared (MIR)-photons released from ATP hydrolysis will be resonantly absorbed by polymerase and break the appointed bond of confined molecules, with high energy efficiency and ultrahigh selectivity. Similarly, this kind of quantized chemical reaction may also happen in P-type ATPase and facilitate ion transport, with the allowance of confined resonance structure. However, if there is no such resonance structure in P-type ATPase, the MIR photons will drive thermal movement of surrounding molecules indiscriminately, which can also facilitate ion transport. In addition, the model introduced in this study only focus on the thermodynamic equilibrium, but not the ion details. As a result, specific amounts or types of ion transport only have contribution to entropy change. In Na-K-ATPase, five cations can be pumped against the concentration gradient, but only one ATP is used for each transport event if $\Phi$ > 0. It should be noted that Na-K pump can still work in the absence of K+. Even if there is K+ exist, the ϵ in Eq.3 will become ϵ^Na^ + ϵ^K^ instead, which has no effect on the following calculation.

**Limitations**

This model introduced in this letter only descripted the pump behavior from the perspective of entropy. It is well acknowledged that the quantum effects dominate the physics behavior in molecules, atoms and subatoms scale. Therefore, quantum effects must exist in the behavior of ions and channels, including but not restricted to electron spin and bond. However, the description of these effects is difficult due to the limited knowledge. Here we temporarily ignore the transmembrane potential, because it has little effect on ion thermodynamic fluctuation on average. Although the movement of charged particles against the potential consumes energy from ATP, the cost won’t affect the dynamic balance. From thermodynamics view, thermal fluctuations are a basic manifestation of the temperature of systems: A system at nonzero temperature does not stay in its equilibrium microscopic state, but instead randomly samples all possible states, with probabilities given by the Boltzmann distribution, as shown in Eq. (1) - (2). Thermodynamic variables, such as pressure, temperature and number of particles N, etc. will affect the probability of fluctuation. Thus, the pump states between ‘f’ and ‘e’ will not be affected by transmembrane potential. For the ion, the adhesion with pump happens in super small scale that the potential can be ignored. The free energy from ATP hydrolysis only allows the ion transport happening when deducts energy consumption from transmembrane potential or conformational changes, from the perspective in this model. From a macro perspective, transmembrane potential ranges from -70 to 40 mV dynamically. As a result, the potential will either push or pull transmembrane transport, which will cancel each other out on average. Even in the hyperpolarized condition (-70 mV), energy cost during transmembrane transport by one positive ion is about 7 kJ/mol, much less than energy release from ATP hydrolysis (30 kJ/mol).

Therefore, in this thermodynamic model, we only talk about the condition that transmembrane potential equals to zero, which is without loss of generality.

Supplementary Fig. 1.


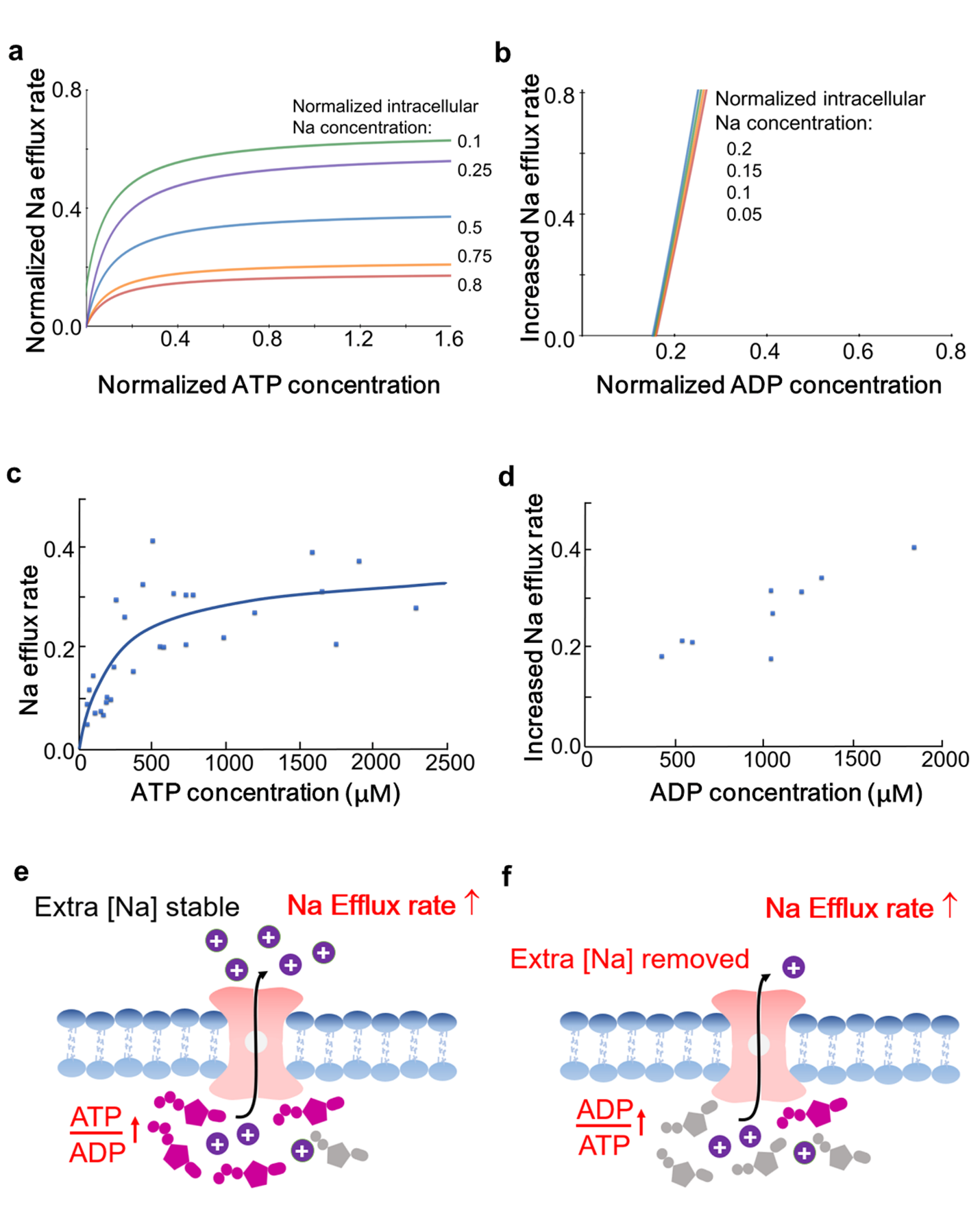


**Supplementary Fig. 1. Na-K exchange rate affected by the intracellular ATP concentration and removal of extracellular Na.** (a) Na efflux rate controlled by increasing ATP concentration at various normalized preset intracellular Na concentrations. Normalized ADP=0.2; extracellular Na=0.8. (b) Increased Na efflux rate after the removal of extracellular Na by increasing ADP concentration at various normalized preset intracellular Na concentrations. Normalized ATP=0.2; extracellular Na=0. (c) The effect of various intracellular ATP levels on ouabain-sensitive Na-K exchange in resealed ghosts. The scatter plot is the result in each experiment, and the curve is the mean value. (d) The stimulation percentage of ouabain-sensitive Na efflux after the removal of external Na by increasing intracellular ADP. The origin Na-containing medium contained 135 mM NaCl, and the Na free medium contained 135 mM choline Cl. Each point represents the percent change in the ouabain-sensitive Na flux components. The phosphoarginine regenerating system was used to set and maintain ADP and ATP concentrations. (e) - (f) Illustrations of these two effects.

**Supplementary References**

1 Kennedy, B. G., Lunn, G. & Hoffman, J. F. Effects of altering the ATP/ADP ratio on pump-mediated Na/K and Na/Na exchanges in resealed human red blood cell ghosts. *J Gen Physiol.* **87**, 47-72 (1986).

2 Hoffman, J. F. The active transport of sodium by ghosts of human red blood cells. *J Gen Physiol.* **45**, 837-859 (1962).

3 Maxwell, J. C. *Theory of heat*. (Longmans, Green and co., 1871).

4 Mandal, D. & Jarzynski, C. Work and information processing in a solvable model of Maxwell's demon. *Proc Natl Acad Sci U S A.* **109**, 11641-11645 (2012).

5 Mandal, D., Quan, H. T. & Jarzynski, C. Maxwell's refrigerator: an exactly solvable model. *Phys Rev Lett.* **111**, 030602 (2013).

6 Seah, S., Nimmrichter, S. & Scarani, V. Maxwell's Lesser Demon: A Quantum Engine Driven by Pointer Measurements. *Phys Rev Lett.* **124**, 100603 (2020).

7 Kunji, E. R. *et al.* The transport mechanism of the mitochondrial ADP/ATP carrier. *Biochim Biophys Acta.* **1863**, 2379-2393 (2016).

8 Schrödinger, E. *What is life? and other scientific essays*. (Doubleday, 1956).

9 Hodgkin, A. L. & Huxley, A. F. A quantitative description of membrane current and its application to conduction and excitation in nerve. *J Physiol.* **117**, 500-544 (1952).

10 Gatenby, R. A. & Frieden, B. R. Information theory in living systems, methods, applications, and challenges. *Bull Math Biol.* **69**, 635-657 (2007).

11 Monti, J. L. E., Montes, M. R. & Rossi, R. C. Steady-state analysis of enzymes with non-Michaelis-Menten kinetics: The transport mechanism of Na(+)/K(+)-ATPase. *J Biol Chem.* **293**, 1373-1385 (2018).

12 Dyla, M. *et al.* Dynamics of P-type ATPase transport revealed by single-molecule FRET. *Nature.* **551**, 346-351 (2017).

13 Li, N. *et al.* Demonstration of biophoton-driven DNA replication via gold nanoparticle-distance modulated yield oscillation. *Nano Res.* **14**, 40-45 (2021).

14 Zhang, F., Song, B. & Jiang, L. The quantized chemical reaction resonantly driven by multiple MIR-photons: From nature to the artificial. *Nano Res.* **14**, 4367-4369 (2021).
